# Foundations of Interprofessional Education in Dental Schools: A Narrative Review

**DOI:** 10.1055/s-0045-1804504

**Published:** 2025-03-12

**Authors:** Ahmed Yaseen Alqutaibi, Mohamed M. Rahhal, Rawda Awad, Omer Sheriff Sultan, Mohamed A. M. Iesa, Muhammad Sohail Zafar, Mohamed Jaber

**Affiliations:** 1 Substitutive Dental Sciences Department (Prosthodontics), College of Dentistry, Taibah University, Al Madinah, Saudi Arabia; 2 Prosthodontics Department, College of Dentistry, Ibb University, Ibb, Yemen; 3 Department of Prosthodontics, Missouri School of Dentistry and Oral Health (MOSDOH), A.T. Still University, Saint Louis, Missouri, United States; 4 Department of Prosthodontics, Faculty of Oral and Dental Medicine, Fayoum University, Fayoum, Egypt; 5 Restorative Dentistry, Missouri School of Dentistry and Oral Health (MOSDOH), A.T. Still University, Saint Louis, Missouri, United States; 6 Restorative Dentistry, Missouri School of Dentistry and Oral Health (MOSDOH), A.T. Still University, Kirksville, Missouri, United States; 7 Department of Physiology, Al Qunfudah Medical College, Umm Al Qura University, Mecca, Saudi Arabia; 8 Department of Clinical Sciences, College of Dentistry, Ajman University, Ajman, United Arab Emirates; 9 Centre of Medical and Bio-allied Health Sciences Research, Ajman, University, Ajman, United Arab Emirates; 10 School of Dentistry, Jordan University, Amman, Jordan

**Keywords:** dental education, interprofessional collaboration, educational environment, IPE curriculum, learning theory

## Abstract

Interprofessional education (IPE) and interprofessional collaborative practice have gained significant recognition for their ability to enhance health care education and improve patient outcomes, particularly in dentistry. Given the close connection between oral and general health, incorporating IPE into dental curricula has become essential in preparing practitioners for collaborative patient-centered care. This review focuses on the foundations of IPE in dental schools, focusing on its role in preparing students for collaborative health care. It discusses the evolution and current trends of IPE, examples of existing programs, and the importance of interprofessional teamwork for dental students. Key interprofessional competencies, such as communication, ethics, and teamwork, are highlighted, along with methods for assessing these skills, emphasizing the value of IPE in developing well-rounded dental professionals.

## Introduction

Interprofessional education (IPE) and interprofessional collaborative practice (IPCP) have emerged as significant educational models aimed at enhancing health care outcomes. These concepts, which date back to the early 20th century, have gained momentum in recent years for their potential to improve health care education and foster better patient care. 1 IPE, in particular, plays a critical role in enhancing patient safety, reducing medical errors, and preparing health care professionals to effectively collaborate in today's complex health care systems. 2 It has become a cornerstone in health care education globally, breaking down the traditional silos between professions such as physicians, nurses, and dentists to promote a culture of collaboration and patient-centered care. However, while IPE and IPCP are often referred to as “game changers” in health care, there is a need for concrete examples and empirical evidence that demonstrate their tangible impact on patient outcomes and educational practices. 2 3

In dentistry, the role of IPCP is particularly important due to the strong link between oral and general health. Dentists and dental hygienists often serve as the first line of defense in detecting underlying systemic conditions, making collaboration with other healthcare system essential. 4 Although these collaborative efforts have the potential to transform oral health care and improve efficiency, the shift toward team-based care is not without its challenges. Breaking down entrenched professional hierarchies, particularly in institutions with deeply rooted traditional practices, can lead to resistance. Additionally, while the Affordable Care Act emphasized the importance of team-based care and facilitated the implementation of Accountable Care Organizations, the impact of such policies on IPE/IPCP adoption across different health care settings varies and warrants further exploration. 3 5

Furthermore, while the literature highlights the benefits of IPE/IPCP, it often overlooks the complexities and limitations associated with these models. For instance, the challenges in implementing IPE in dental education, such as curriculum integration and faculty development, are underexplored. There is also a gap in understanding the long-term sustainability and effectiveness of IPE in improving patient outcomes and fostering interprofessional collaboration. Although dental education has embraced IPE as a tool for improving communication skills, teamwork, and holistic care, the empirical data supporting these outcomes remains sparse and inconclusive. 3

The Interprofessional Education Collaborative (IPEC), founded in 2009 in the United States, has significantly advanced IPE for health care professions education. The American Dental Education Association, a key U.S.-based member of IPEC, actively encourages the integration of IPE in dental academic institutions and augments collaboration with other health care professions' programs to enhance oral health care and outcomes. 6 Notably, 82% of graduating dental students in the United States reported that engaging with diverse health professions during their academic journey improved their ability to provide enhanced patient care.

The Commission on Dental Accreditation (CODA), the accrediting body for dental education programs in the United States, has emphasized the importance of IPE in preparing dental students to work in collaborative health care environments. CODA's standards have played a crucial role in shaping how dental programs integrate IPE into their curricula to promote comprehensive patient care. In addition, IPE/IPCP empowers dental students to enrich their education and motivates them to become leaders in advocating for collaborative practice and team-based care. 7 8 The IPE/IPCP has diverse advantages for health care students. First, these approaches facilitate a platform for students to refine their communication skills by connecting with peers from different health care fields. This interaction cultivates an appreciation for the unique terminologies used in other professions and underscores the paramount importance of clear and concise communication. Second, IPE/IPCP fosters a deeper understanding of the roles and responsibilities of various health care professionals, which is pivotal for effective teamwork and the delivery of comprehensive patient care. Lastly, the collaborative nature of IPE/IPCP encourages students to work collectively in addressing complex health issues, also providing valuable insights into the collaborative dynamics that define the health care environment. 9 Despite IPE's significant role in the professional development of dental students for collaborative health care practice and enhancing patient outcomes, empirical data regarding its long-term sustainability is sparse. Furthermore, the existing evidence on the effectiveness of IPE in teaching interprofessional communication skills, achieving patient care objectives, and imparting clinical skills is inconclusive and requires further investigations. 10 Therefore, this review aims to explore the foundational aspects of IPE within dental schools, emphasizing its significance and applicability in preparing dental students for collaborative health care environments. It begins by defining IPE and its critical role in health care, highlighting how it supports the distinct needs of dental students by fostering a collaborative mindset early in their training. A broad overview is presented, tracing the evolution of IPE in dental education, identifying current trends, and examining specific programs implemented in dental schools. Further, the review underscores the importance of interprofessional collaboration, detailing the value of teamwork across health care disciplines and the positive impact of such collaborative learning on dental students. Additionally, it outlines essential interprofessional competencies for dental students, particularly in areas like communication, ethics, and teamwork, and explores various assessment methods used to measure these competencies effectively. Through this structured approach, the review aims to provide insights into how IPE can be integrated into dental curricula to produce well-rounded professionals prepared for multidisciplinary health care settings.

## Methods

A thorough examination of relevant peer-reviewed literature was undertaken across various databases, including PubMed, Scopus, Web of Science, and Google Scholar. The search utilized keywords such as “interprofessional education,” “dental education,” “healthcare collaboration,” “IPE integration,” and “dental curricula.” Boolean operators (AND, OR) were employed to refine the search and enhance the comprehensiveness of the included studies. The selected articles for review were published in English and concentrated on IPE within dental schools, frameworks for the integration of IPE, and the impact of IPE on dental students and multidisciplinary teams.

## Overview of Interprofessional Education in Dental Schools

Before embarking on the development and implementation of IPE within institutional settings, it is imperative to delineate the constituent components of IPE rigorously. In 2010, the World Health Organization (WHO) formulated a comprehensive “Framework for Action on Interprofessional Education and Collaborative Practice.” According to the WHO, IPE is defined as a pedagogical paradigm wherein students from two or more distinct professions engage in reciprocal learning experiences to foster effective collaboration and enhanced health outcomes. Additionally, the WHO expounds upon IPCP as the orchestrated collaboration of multiple health practitioners, stemming from diverse professional backgrounds, working in concert with patients, families, caregivers, and communities to achieve the pinnacle of health care delivery.

Given the escalating intricacies inherent in the contemporary health care landscape, the imperative for collaborative practice has become paramount. The integration of IPE and IPCP is a crucial pathway, systematically endowing students with the requisite knowledge and skills for imminent collaborative undertakings in the dynamic health care milieu. In 2011, the IPEC, a consortium representing educational institutions in health professions, introduced a set of shared core competencies applicable to all health care disciplines. 11 These competencies are strategically crafted to equip health care professionals with the requisite interprofessional collaboration skills. The composition of the expert panel tasked with formulating these competencies reflects a diverse spectrum of educators spanning fields such as medicine, dentistry, nursing, osteopathic medicine, pharmacy, and public health. The primary objective underlying these competencies is to furnish health care professionals with the essential capabilities to engage in purposeful and effective collaboration, ultimately contributing to establishing a safer, patient-centered, and community-focused health care system. Notably, these core competencies are designed to complement the discipline-specific proficiencies of each profession, catering to the educational needs of students and seasoned practitioners. The expert panel delineated four domains of interprofessional competency, each accompanied by a comprehensive statement and a set of specific behavioral subcompetencies. 12 These subcompetencies function as quantifiable benchmarks, representing milestones that learners should attain by the culmination of their educational journey, preceding licensure, or certification.

The four interprofessional core competencies, along with their corresponding general competency statements updated in 2016, are as follows 12 :

1. Values/Ethics for Interprofessional Practice: Collaborate with individuals from diverse professions to cultivate an environment characterized by mutual respect and shared values.
2. Roles/Responsibilities: Employ knowledge about one's role and the roles of other professions to judiciously assess and address the health care needs of patients and populations.
3. Interprofessional Communication: Engage in responsive and responsible communication with patients, families, communities, and fellow health professionals, fostering a team-oriented approach to health maintenance and disease treatment.
4. Teams and Teamwork: Apply relationship-building values and principles of team dynamics to proficiently function in varied team roles, facilitating the planning and delivery of patient/population-centered care characterized by safety, timeliness, efficiency, effectiveness, and equity.

Understanding the essence of IPE entails the recognition of its boundaries and principles. It extends beyond the mere physical copresence of students from various health professions, where they passively receive identical information. True IPE necessitates active, reflective interaction among students to foster collaborative learning and develop a mutual understanding of the roles of each profession involved. Furthermore, effective IPE surpasses the inclusion of faculty members from diverse professions leading classes; it involves illustrating how these professions interact within health care contexts to facilitate interprofessional learning experiences. 13 14 Furthermore, IPE is not fulfilled by merely participating in a patient care setting led by a professional from another field without shared decision-making or shared responsibility for patient care. True IPE embraces an integrated approach where health care professionals collaborate in the care process, ensuring the patient's well-being is at the center of their collective efforts. 15

Academic dental institutions often incorporate IPE and team-based care initiatives into their curricula, enabling students to gain early exposure to interprofessional teams. This pedagogical approach empowers dental students with collaborative skills and a comprehensive perspective on patient care that extends beyond oral health. This transformative approach ensures dental graduates are well-prepared to collaborate with other health care providers, contributing to enhanced health care delivery, improved patient outcomes, and, ultimately, the overall health of individuals and communities. IPE provides dental students with the understanding that they are integral members of the health care team, and their contributions extend beyond the oral cavity to impact the holistic health of their patients. 16

In conjunction with other health profession programs, health center schools actively advocate for integrating collaborative learning experiences into their educational curricula. The primary objective is to ensure that graduates possess a comprehensive understanding of various health professions' roles and responsibilities, emphasizing teamwork's positive impact on patient care. This shift from classroom-based learning to practical clinical experience underscores the growing importance of interprofessional collaboration. In this context, learning becomes more relationship-oriented, featuring intricate interactions with diverse stakeholders, including patients, families, and communities. IPE can manifest at any stage along the education-to-clinical-practice continuum, whether formally structured or informally integrated. Formal IPE activities play a pivotal role in developing collaborative competence, often of didactic or simulation-based activities that frequently occur in closely supervised clinical settings. 17

IPE in dental programs confers a variety of advantages, foremost among them being the enhancement of patient-centric care. Dental students exposed to IPE and IPE acquire the ability to function with multidisciplinary teams, emphasizing holistic patient well-being. This exposure facilitates insights into effective coordination with other health care professionals, fostering comprehensive and patient-centered treatment approaches. Moreover, IPE enhances dental students' communication skills, which is critical for effective collaboration within diverse healthcare. The emphasis on teamwork also enriches interpersonal skills and broadens their knowledge base. Exposure to various health care disciplines deepens dental students' understanding of the roles and responsibilities of other professionals, enabling informed decision-making. Additionally, IPE equips students to tackle complex cases comprehensively, exploring diverse treatment options. The focus on professionalism, ethics, and cultural competence further enhances dental students' understanding of ethical principles and cultural sensitivity. 18 19 20 21

The integration of IPE encounters barriers across different organizational levels, which may impact administration, faculty members, and students. Key obstacles to IPE include scheduling conflicts, rigid curricula, interprofessional conflicts, and a perceived lack of values. In addition, attitudinal differences among health care professionals, faculty, and students also affect IPE implementation, while a scarcity of resources and lack of commitment can impede its success. 15 Administrative challenges involve concerns about resource allocation and competing institutional demands. Reallocating resources to accommodate changes in health care education is crucial. Addressing logistical issues, such as scheduling and classroom space, is vital at the administrative level to establish sustained IPE commitment. Faculty members must appreciate the benefits of IPE and actively participate despite potential resistance. Leadership within the professional field should motivate faculty members and implement systems to reward efforts in developing and integrating IPE into the curriculum. Operational aspects must align to support IPE, including physical space, course design, and scheduling. 15

In dental education, incorporating IPE presents challenges, including coordinating schedules and aligning curricula across various health professions programs, resource allocation for faculty training and infrastructure development, and assessing effectiveness across diverse programs. Resistance to change from faculty and students may hinder integration, necessitating a shift in teaching methods. Curriculum integration and the heterogeneity of student groups pose fundamental challenges. Navigating accreditation standards and professional licensing requirements adds complexity. Despite these barriers, overcoming challenges is essential for the comprehensive integration of IPE into health care education. 15 22 The integration of interprofessional clinical experiences in health care education manifests through various methodologies, underscoring the dynamic evolution of IPE. Numerous approaches 23 24 25 have been documented, each presenting distinctive advantages and insights conducive to fostering interprofessional collaboration and facilitating holistic patient care.

1. Colocation of clinics 26 : This approach entails situating clinics from different health care professions in close proximity. The physical adjacency fosters seamless interactions and collaboration among diverse health care disciplines. This environment facilitates mutual learning among students, enabling them to observe the interconnectedness of their respective roles in patient care.
2. Integration of professional services 27 : Some institutions integrate services provided by other health care professions into the dental school clinic. This immersive approach exposes dental students to a multidisciplinary environment where they collaborate with professionals from various fields, such as nursing or pharmacy. Consequently, students acquire a comprehensive understanding of patient care that extends beyond the confines of their specific discipline.
3. Community-based clinical experiences 28 : Interprofessional clinical experiences can extend to community settings, enabling students to engage with diverse patient populations and health care providers. In these settings, students learn to adapt to the specific needs of the community but also collaborate with a spectrum of health care professionals to address complex health care challenges.
4. Interprofessional student–faculty teams 29 : A more hands-on approach involves integrating interprofessional student–faculty teams within the dental school clinic. In this model, students from different health care disciplines collaborate with faculty members, delivering care as a unified team. This approach promotes collaborative learning, with faculty members serving as mentors and guiding students in providing patient-centered care.

These diverse approaches underscore the evolving landscape of IPE, accentuating the significance of teamwork, communication, and collaboration in training future health care professionals. While the field continues to mature, these varied approaches offer invaluable insights into the practical implementation of interprofessional clinical experiences and their potential to reshape health care education. 23

## Interprofessional Collaboration in Dental Education

Collaborative practice in dentistry holds a pivotal role in delivering comprehensive health care. It is fundamentally important as it allows for a holistic approach to patient well-being, addressing oral health and the broader spectrum of general and systemic health. This interdisciplinary collaboration brings together dental professionals, physicians, nurses, pharmacists, and various other health care providers. Effective collaboration allows healthcare professionals to better identify, prevent, and manage health issues. 9 Another key advantage of collaborative practice in dentistry is the enhancement of treatment planning. The collective expertise of various health care professionals ensures that patients receive personalized and well-coordinated treatment plans tailored to meet each patient's unique needs, considering their oral and systemic health. This results in improved patient care effective and efficient patient care. 30 An overview of collaborative practices in dentistry and IPE is presented in Table 1 . The collaborative practice in dentistry is crucial for delivering comprehensive health care, addressing oral and systemic health, and involving various health care professionals. Collaborative practice enhances treatment planning by pooling expertise, leading to personalized and coordinated care plans tailored to individual patient needs. In addition, it plays a crucial role in educating patients about the relationship between oral and systemic health, improving health literacy, and promoting proactive health management. This approach optimizes resource utilization by pooling expertise, minimizing waste, and improving patient care outcomes, while also encouraging innovation and interdisciplinary research. Table 1 provides an overview of these aspects.

**Table 1:** Overview of collaborative practice in dentistry and interprofessional education

Aspect	Summary
Importance of collaborative practice	Collaborative practice in dentistry is crucial for delivering comprehensive health care, addressing oral and systemic health, and involving various health care professionals
Enhancement of treatment planning	Collaborative practice enhances treatment planning by pooling expertise, leading to personalized and coordinated care plans tailored to individual patient needs
Role in patient education	It is crucial in educating patients about the relationship between oral and systemic health, improving health literacy, and promoting proactive health management
Efficient resource utilization	Collaborative practice optimizes resource utilization by pooling expertise, minimizing waste, and improving patient care outcomes
Promotion of interdisciplinary research	It encourages innovation and the development of comprehensive health care solutions by bringing together experts from various fields
Addressing health disparities	Collaborative practice ensures underserved populations receive necessary health care, reducing disparities in access and outcomes
Examples of integrating IPE	Various health professions curricula integrate core interprofessional education competencies, fostering collaboration and communication among health care professionals

Abbreviation: IPE, interprofessional education.

Collaborative practice also plays a crucial role in patient education. Patients benefit from a deeper understanding of the intricate relationship between oral and systemic health. Individuals become aware of how oral health can impact overall well-being and vice versa, leading to improved health literacy and proactive health management. Efficient resource utilization is another facet of the importance of collaborative practice. By pooling the expertise of different health care professionals, resources can be utilized more effectively, optimizing patient care outcomes, and minimizing waste. 2 Furthermore, collaborative practice promotes interdisciplinary research. It encourages innovation and the development of comprehensive health care solutions. This research-driven approach leads to advancements in patient care and overall health care practices. Addressing health disparities is yet another significant benefit of collaborative practice. It ensures that underserved and marginalized populations receive the required health care services, reducing inequalities in health care access and outcomes. 30

Examples of combining core competencies of IPE and collaborative practice can be observed in various health professions curricula. For instance, the CODA has applied new accreditation standards combining the fundamentals of IPE and IPCP into dental programs. These standards require dental graduates to be proficient in communication and collaboration with other health care professionals, prompting dental educators to incorporate IPE and its assessment into their institutions. 31 32 These educational approaches, drawn from diverse health professions curricula, align with the philosophy advocated in the WHO report from the IPEC. This philosophy encourages collaborative designs for educating and training future health care professionals within an ever-evolving health care system. 33 An intriguing observation surfaced in a comprehensive study involving medical, nursing, pharmacy, dental, physician assistant, and physical therapy students: dental students exhibited a less positive outlook on IPE than their medical counterparts. Concurrently, the study unveiled nuanced aspects of in-group identification among nursing and medical students. While nursing students displayed more positive attitudes toward IPE when aligning with their in-group, the opposite was true for medical students. This distinction underscores the importance of understanding dental students' perspectives and engagement in IPE relative to their peers from diverse health professions. These insights into the multifaceted realm of IPE can inform tailored educational strategies to enhance its effectiveness across various health care disciplines. 34

Furthermore, another study at the Tokyo Medical and Dental University delved into the readiness of dental, medical, and nursing students for interprofessional learning before and after participating in IPE workshops. Results indicated significant improvements in readiness across all disciplines following the workshops, with dental students consistently scoring lower than their peers. Qualitative insights provided by dental students shed light on their limited perception of the need for interprofessional collaboration, the belief that dentistry often operates independently, and a sense of insufficient contribution to the workshops. These findings underscore the imperative to enhance IPE programs, foster interprofessional collaboration within dental education, heighten educator awareness, and refine workshop facilitation techniques to address the unique dynamics surrounding dental students' engagement in IPE. 35 A study aimed to assess the effectiveness of an 8-week pilot oral health interprofessional program involving students from dentistry, medicine, nursing, and pharmacy disciplines, particularly in addressing the oral health needs of disadvantaged elderly populations. The program was designed based on pedagogical principles emphasizing care, critical thinking, communication, and collaboration, aligned with the 4Ms model. It featured four scenarios of dental complications in the elderly, including Alzheimer's disease, oral cancer, Parkinson's disease, and stroke. A mixed-methods evaluation was conducted, and the results revealed a significant improvement in students' knowledge and attitudes, with increased confidence in practicing within the age-friendly health system. The study highlighted IPE's role in improving students' awareness of diverse health care services and hands-on learning through scenario-based training, especially in geriatric care. 36

A University of California, Los Angeles School of Dentistry study evaluated the impact of the Strategic Partnership for Interprofessional Collaborative Education in Pediatric Dentistry (SPICE-PD) program on pediatric and general dentistry residents, pediatric medical residents, and pediatric nurse practitioner (PNP) students. A survey of 208 participants showed that SPICE-PD improved interprofessional collaboration and understanding of health care roles. Pediatric medical residents and PNPs gained skills in early childhood caries screening, with PNPs showing significant improvement in fluoride varnish application. Most recognized the importance of integrating oral health into patient care and facilitating dental referrals, highlighting the potential of IPE programs like SPICE-PD to enhance oral health and comprehensive care. 37

The “Collaborative Home for Oral Health, Medical Review, and Health Promotion” (CHOMP) program at Case Western Reserve University, supported by a grant from the Health Resources and Services Administration, aims to promote IPE and collaborative care. This program involves teams with nurse practitioner (NP) students, NP faculty members, dental students, and dental faculty members. In this model, dental and NP students collaborate in gathering patient health information and conducting basic tests, all under the supervision of faculty from both professions. The program encourages shared decision-making, increases access to primary care, and enhances student collaboration skills. Data on billable services NPs provide is being collected to assess the program's sustainability. Dental students play a vital role in this collaborative care approach, actively contributing to patient care planning and gaining valuable insights into health care teamwork. 23 Dalhousie University's “Seamless Care” model places students from various health disciplines in community settings to improve collaborative skills through hands-on experience. Teams from dentistry, medicine, nursing, and pharmacy assist patients transitioning from hospital to community, guided by faculty and professionals. The 8-week placement includes orientation and mentoring, with a focus on patient-centered care. Supported by a Health Canada grant, the model is based on social learning theory, emphasizing learning through observation and role modeling. This approach enhances both student skills and patient care. 23

The University at Buffalo has integrated social work services into its dental clinic to address access barriers for underserved populations. In 2001, the institution established the Counseling, Advocacy, Referral, Education, and Services (CARES) program, which focuses on improving oral health by reducing barriers and enhancing access to dental treatment for individuals with special needs and those who are difficult to reach. Dental students participating in the program utilize a screening protocol to identify patients who encounter challenges in returning for dental treatment. The interprofessional approach employed in this clinical setting enriches students' understanding of the advantages of a multidisciplinary approach to patient care. Although dental and social work students do not interact with patients simultaneously, the coordination of services supports a patient-centered approach. This method plays a significant role in addressing both health and social barriers, thereby improving access to dental services. Dental students are pivotal in this collaborative model; they gain valuable experience while contributing to the program's overall success. In 2009, 45% of CARES participants reported that they would not have completed their dental care without the program's intervention. 23

At New York University (NYU), an innovative IPE approach colocates multiple health profession clinics. In 2005, a partnership between NYU's College of Dentistry and College of Nursing led to the opening of a Nursing Faculty Practice facility within the dental school. Dental and dental hygiene students identify patients needing primary care and refer them to NPs at this facility. Dental and NP students collaborate during clinical rotations to address both oral and systemic health issues. Additionally, NYU's College of Nursing launched the Oral Health Nursing Education and Practice program to raise awareness of oral health among nurses and improve referral practices. 23

## Interprofessional Competencies for Dental Students

Five overarching themes comprise the IPE essential competencies 38 :

1. Roles and responsibilities
2. Ethical practice
3. Conflict resolution
4. Communication
5. Collaboration and teamwork

When formulating an interprofessional activity, at least one theme should be considered as a potential outcome. Whenever feasible, these themes should be aligned with an assessment task. 38 39 40 An overview of key considerations in designing and implementing IPE curricula is presented in Table 2 .

**Table 2:** Overview of key considerations in designing and implementing interprofessional education curricula

Aspects considered	Details
IPE essential competencies	Roles and responsibilities, ethical practice, conflict resolution, communication, collaboration and teamwork
Learning objectives	Attitudes, knowledge, abilities, professional conduct
Curriculum development	Standardized educational outcomes, modification of attitudes, knowledge, and behaviors, consensus-building, curriculum structure and course development
Planning and execution	Objective setting, shared philosophy determination, model framework construction, phased approach implementation
Assessment strategy	Assessment plan development, cross-professional assessment committee, quantitative instruments selection, mixed-method evaluation consideration
Curriculum framework	Unified philosophy, theoretical and curricular framework establishment, diverse pedagogical approaches, visual representation utilization
Learning methods	Simulation, case-based learning, experiential learning, technology-enhanced learning (TEL), peer learning
Instructional strategies	Utilization of prior knowledge, facilitation of self-evaluation, peer learning encouragement, simulation utilization
Learning theories	Self-efficacy, communities of practice, reflective learning

Abbreviation: IPE, interprofessional education.

Using a structured and planned learning experience, IPE aims to develop the attitudes, knowledge, abilities, and professional conduct of students. 41 In developing interprofessional activities, constructive alignment guarantees that learning objectives are precisely matched with the activity itself and pertinent assessment tasks. Establishing this for the participating pupils at the onset of an activity is imperative. The academic and practical features of leading groups of students from different professions are similar to those of leading groups of students from a single profession; activity design and planning remain the same. Research suggests, however, that facilitators need to modify their teaching strategies to engage students and direct their learning concerning different professions. Consequently, this necessitates more stringent preparation and direction. 42 Dental students must comprehend the correlation between oral health and overall health to achieve more resolute, patient-centered, and secure care. The IPE provides the knowledge and experience necessary for students to attain this comprehension. It is strongly suggested that it be integrated into dental curricula. 43 44 45

## Conclusion

The integration of IPE within dental schools offers transformative potential in preparing dental students for collaborative, patient-centered health care. By embedding core interprofessional competencies, such as communication, ethics, teamwork, and an understanding of diverse roles, IPE aligns dental education with the demands of modern health care, where comprehensive patient care requires multidisciplinary efforts. IPE enhances students' ability to work effectively in interprofessional teams, reinforcing the importance of holistic health that extends beyond dental care.

Looking forward, the continued evolution of IPE in dental education will require addressing persistent challenges, such as aligning curricula across health professions, developing shared assessment strategies, and fostering a cultural shift among faculty and students toward collaborative learning. Future directions could focus on expanding community-based and experiential learning opportunities, increasing support for interprofessional faculty development, and leveraging technology to facilitate cross-disciplinary learning. These advancements will ensure that IPE prepares students for effective team-based care and contributes to building a healthcare system that is safe, efficient, and focused on the comprehensive health needs of individuals and communities.
